# Supplementary material for: Meta-Analysis and Gene Set Enrichment Relative to ER Status Reveal Elevated Activity of MYC and E2F in the “Basal” Breast Cancer Subgroup
Source: PLoS One. 2009 Mar 9;4(3):e4710. doi: 10.1371/journal.pone.0004710 (PMC2650420; doi:10.1371/journal.pone.0004710)
Supplement: Dataset S1 — GSEA results from first screen. The file contains a zipped archive of the HTML results of the second GSEA screen. Reports for enriched or depleted datasets may be accessed from the “index.html” link within the my_analysis.GseaPreranked.1219975950625.rpt.zip ZIP archive. Enrichment plots are available for each of the 50 most enriched and depleted gene sets. (13.32 MB ZIP) [file pone.0004710.s001.zip › my_analysis.GseaPreranked.1219975950625.rpt.zip!//AGED_MOUSE_HIPPOCAMPUS_ANY_UP.html]

Details for gene set AGED\_MOUSE\_HIPPOCAMPUS\_ANY\_UP[GSEA]

|  || Dataset | zSco.ERneg.to.ERpos\_collapsed |
| Phenotype | NoPhenotypeAvailable |
| Upregulated in class | na\_neg |
| GeneSet | AGED\_MOUSE\_HIPPOCAMPUS\_ANY\_UP |
| Enrichment Score (ES) | -0.50029343 |
| Normalized Enrichment Score (NES) | -1.919108 |
| Nominal p-value | 0.002793296 |
| FDR q-value | 0.014538525 |
| FWER p-Value | 0.38 |
Table: GSEA Results Summary

  

Fig 1: Enrichment plot: AGED\_MOUSE\_HIPPOCAMPUS\_ANY\_UP      
 Profile of the Running ES Score & Positions of GeneSet Members on the Rank Ordered List

  

| PROBE | GENE SYMBOL | GENE\_TITLE | RANK IN GENE LIST | RANK METRIC SCORE | RUNNING ES | CORE ENRICHMENT || 1 | DDIT4 |  |  | 1085 | 3.375 | -0.0409 | No |
| 2 | SMARCC1 |  |  | 1812 | 2.684 | -0.0631 | No |
| 3 | EIF2S2 |  |  | 2127 | 2.437 | -0.0573 | No |
| 4 | ENPP2 |  |  | 2229 | 2.366 | -0.0363 | No |
| 5 | TIA1 |  |  | 2434 | 2.233 | -0.0247 | No |
| 6 | KCNA1 |  |  | 2559 | 2.169 | -0.0078 | No |
| 7 | MEF2C |  |  | 2965 | 1.952 | -0.0147 | No |
| 8 | CTSZ |  |  | 4186 | 1.438 | -0.0892 | No |
| 9 | KCNB1 |  |  | 4526 | 1.310 | -0.0989 | No |
| 10 | COL6A1 |  |  | 5465 | 0.987 | -0.1576 | No |
| 11 | SCN1A |  |  | 5485 | 0.978 | -0.1472 | No |
| 12 | KIF5B |  |  | 5668 | 0.924 | -0.1498 | No |
| 13 | PENK |  |  | 6246 | 0.731 | -0.1844 | No |
| 14 | GPD2 |  |  | 6605 | 0.618 | -0.2039 | No |
| 15 | GNA13 |  |  | 6893 | 0.521 | -0.2192 | No |
| 16 | PTEN |  |  | 7664 | 0.243 | -0.2742 | No |
| 17 | SLC4A5 |  |  | 7845 | 0.170 | -0.2857 | No |
| 18 | CRY1 |  |  | 8208 | 0.032 | -0.3126 | No |
| 19 | DBP |  |  | 8458 | -0.058 | -0.3306 | No |
| 20 | MARCH7 |  |  | 8751 | -0.188 | -0.3504 | No |
| 21 | TXNIP |  |  | 9553 | -0.592 | -0.4035 | No |
| 22 | PBX3 |  |  | 10267 | -1.001 | -0.4451 | No |
| 23 | RBM3 |  |  | 11001 | -1.566 | -0.4814 | Yes |
| 24 | WDR26 |  |  | 11003 | -1.570 | -0.4625 | Yes |
| 25 | IGF1R |  |  | 11217 | -1.756 | -0.4573 | Yes |
| 26 | PCGF2 |  |  | 11264 | -1.799 | -0.4390 | Yes |
| 27 | RBM39 |  |  | 11880 | -2.419 | -0.4561 | Yes |
| 28 | ADIPOR2 |  |  | 12026 | -2.591 | -0.4357 | Yes |
| 29 | FTS |  |  | 12152 | -2.782 | -0.4114 | Yes |
| 30 | ZNF403 |  |  | 12199 | -2.840 | -0.3806 | Yes |
| 31 | SNX17 |  |  | 12243 | -2.903 | -0.3487 | Yes |
| 32 | VTI1B |  |  | 12342 | -3.009 | -0.3197 | Yes |
| 33 | IGFBP6 |  |  | 12398 | -3.098 | -0.2864 | Yes |
| 34 | PPP2R5E |  |  | 12502 | -3.263 | -0.2547 | Yes |
| 35 | FOXP1 |  |  | 12538 | -3.317 | -0.2173 | Yes |
| 36 | NRIP1 |  |  | 12554 | -3.341 | -0.1780 | Yes |
| 37 | TAC1 |  |  | 12571 | -3.372 | -0.1385 | Yes |
| 38 | APOD |  |  | 12582 | -3.398 | -0.0982 | Yes |
| 39 | MAPT |  |  | 12626 | -3.480 | -0.0593 | Yes |
| 40 | ALCAM |  |  | 12765 | -3.783 | -0.0240 | Yes |
| 41 | CIRBP |  |  | 13206 | -5.436 | 0.0086 | Yes |
Table: GSEA details [plain text format]

  

Fig 2: AGED\_MOUSE\_HIPPOCAMPUS\_ANY\_UP: Random ES distribution      
 Gene set null distribution of ES for **AGED\_MOUSE\_HIPPOCAMPUS\_ANY\_UP**
